# TCLP: an online cancer cell line catalogue integrating HLA type, predicted neo-epitopes, virus and gene expression

**DOI:** 10.1186/s13073-015-0240-5

**Published:** 2015-11-20

**Authors:** Jelle Scholtalbers, Sebastian Boegel, Thomas Bukur, Marius Byl, Sebastian Goerges, Patrick Sorn, Martin Loewer, Ugur Sahin, John C. Castle

**Affiliations:** TRON – Translational Oncology at the University Medical Center of Johannes Gutenberg University, Freiligrathstrasse 12, 55131 Mainz, Germany; University Medical Center of the Johannes Gutenberg-University Mainz, 55131 Mainz, Germany; Biopharmaceutical New Technologies (BioNTech) Corporation, An der Goldgrube 12, 55131 Mainz, Germany; Present address: European Molecular Biology Laboratory, Meyerhofstraße 1, 69117 Heidelberg, Germany; Present address: Agenus and 4-Antibody AG, Hochbergerstrasse 60C, CH-4057 Basel, Switzerland

## Abstract

Human cancer cell lines are an important resource for research and drug development. However, the available annotations of cell lines are sparse, incomplete, and distributed in multiple repositories. Re-analyzing publicly available raw RNA-Seq data, we determined the human leukocyte antigen (HLA) type and abundance, identified expressed viruses and calculated gene expression of 1,082 cancer cell lines. Using the determined HLA types, public databases of cell line mutations, and existing HLA binding prediction algorithms, we predicted antigenic mutations in each cell line. We integrated the results into a comprehensive knowledgebase. Using the Django web framework, we provide an interactive user interface with advanced search capabilities to find and explore cell lines and an application programming interface to extract cell line information. The portal is available at http://celllines.tron-mainz.de.

**Electronic supplementary material:**

The online version of this article (doi:10.1186/s13073-015-0240-5) contains supplementary material, which is available to authorized users.

## Background

Cancer cell lines are important tools for cancer and immunological research [1–3] and are thus used daily in laboratories and manufacturing. While genomic and immunological characterization of these cell lines is essential, publicly available information is far from complete and typical lab assays are expensive and laborious. Furthermore, most annotations have not used ontologies or controlled vocabularies. Thankfully, due to efforts made by others, such as the Cancer Cell Line Encyclopedia (CCLE) [4] and Klijn *et al.* [5], many cell lines have been sequenced, mutations have been annotated, and raw datasets made publicly available.

We have developed bioinformatics workflows capable of using these datasets to further annotate each cell line, including the cell line origin, 4-digit HLA types [6], gene expression levels, expressed viruses, and mutations. Somatic tumor mutations that give rise to mutated antigens presented on the cell surface (neo-epitopes) are potent targets for cancer immunotherapy [1, 3]. The number of neo-antigens are further associated with the overall survival of cancer patients [7] and the clinical response to CTLA-4 and PD-1 checkpoint blockade in melanoma patients [8–10]. Here, we integrated the cell line-specific mutation information with the determined cell line-specific HLA types and HLA binding prediction algorithms to generate a catalog of cell line-specific predicted HLA Class I and Class II neo-antigens.

Not only are these underlying characterizations important, but also the ability to easily query them in an effective user interface is similarly essential. For example, easy identification of a cell line appropriate for a specific experiment would be enabling, such as quickly filtering for a cell line with a specific HLA type and a specific gene expression. Here, we address these challenges by re-analyzing RNA-Seq data of 1,082 cancer cell lines and integrating all results and available annotation in a centralized cell line annotation database and user-friendly interface, called the TRON Cell Line Portal (TCLP). To our knowledge, the TCLP is the largest catalog of cancer cell line annotations integrating HLA type, HLA expression, predicted HLA Class I and Class II neo-epitopes, virus, and gene expression.

## Construction and content 

All the datasets integrated into the TCLP are publically available: we downloaded the raw data and meta-data annotations, assigned each sample name using a controlled vocabulary (that is, tissue ontology) and processed the associated next generation sequencing (NGS) reads using a computational workflow comprising gene expression analysis; virus identification; determination of HLA type and HLA expression; neo-epitope prediction based on cell line-specific nucleotide mutations, determined HLA type and HLA binding prediction algorithms. The resultant characterizations are loaded into a database, accessible through a web-based user interface and API.

### Datasets

#### RNA-Seq datasets

We integrated cancer cell line RNA-Seq data from two sources: The Cancer Cell Line Encyclopedia (CCLE) and Klijn *et al.* [5] (Table 1). CCLE sequenced the transcriptomes of 781 cancer cell lines using 101 nt paired-end sequencing on Illumina HiSeq2000 and HiSeq2500 instruments (https://cghub.ucsc.edu/datasets/ccle.html). Using the GeneTorrent client software (https://cghub.ucsc.edu/software/downloads.html) and the dataset identifiers provided on CGHub, we downloaded aligned paired-end RNA-Seq samples in the Binary Alignment/Map (BAM) format [11]. Using the Picard BAM2FASTQ tool (http://picard.sourceforge.net), we converted the downloaded BAM files to FASTQ for further processing. Klijn *et al.* [5] analyzed the transcriptional landscape of 675 human cancer cell lines, using 75 nt paired-end sequencing on an Illumina HiSeq 2000 instrument. After gaining access, we downloaded the raw RNA-Seq data in FASTQ format from the European Genome-phenome archive, accession EGAD00001000725 (https://www.ebi.ac.uk/ega/datasets/EGAD00001000725).Of the 675 cell lines, 374 overlapped with the CCLE samples and thus we only processed the unique 301 cancer cell lines.

**Table 1:** External data processed and integrated into the cell line portal

Data type	Source	Number cell lines	Reference
Cancer cell line RNA-Seq data (2 × 101 bp)	CCLE	781	[4], https://cghub.ucsc.edu/datasets/ccle.html
Cancer cell line RNA-Seq data (2 × 75 bp)	Klijn *et al.*	301	[5], https://www.ebi.ac.uk/ega/datasets/EGAD00001000725
Mutations	CCLE	781	[4]
Mutations	Klijn *et al.*	675	[5], Supplementary data 3
HLA Class I and Class II types	Adams *et al.*	49	[16]

#### Mutation and cell line information

We retrieved the cell line annotation, including name, disease, tissue, and mutation information (timestamp 2012.05.07) from the Broad-Novartis Cancer Cell Line Encyclopedia [4] website as well as from Supplementary Data 3 in Klijn *et al.* [5] (Table 1).

### Cell line naming

Sample naming is critical to limit confusion. We store and present the cell line primary name and, following the CCLE naming convention, strip the name of any special characters and convert it to uppercase during processing. To increase the usability of the advanced search, we manually compared and mapped the tissue annotations and disease terms to the corresponding terms from the National Cancer Institute (NCI) Thesaurus (http://ncit.nci.nih.gov/).

### Gene expression

The raw reads were aligned using the STAR algorithm (version 2.3.0e) [12] to the human reference genome (hg19), allowing a total of 2 % mismatches based on read length within the matched sequence. Other settings of STAR remained at default settings. Sequence reads in the resultant alignment files are input into our RNA-Seq analysis, intersected with a BED file containing exons from the UCSC known genes reference table [13], and assigned to the overlapping gene. To calculate gene-level expression, an isoform-to-gene dictionary is used during this process such that if one read overlaps with more than one isoform of a gene, it is counted only once. If the read-to-gene assignment is ambiguous, the count values for the potential genes are all incremented independently. After quantification, the read counts are then normalized to reads per kilobase of exon per million mapped reads (RPKM) [14].

### Virus detection

Reads that did not map to the human genome were aligned to a reference database containing 5,006 virus sequences retrieved from the NCBI Viral Genomes homepage on 29 November 2013 (http://www.ncbi.nlm.nih.gov/genome/viruses/). To detect expressed viruses using the RNA-Seq reads, we recorded the percentage of a virus genome covered by uniquely mapped reads. Over all virus genomes in all reported cell lines we calculated the sum of the mean coverage and the double standard deviation. We used a cutoff of 30 % genome coverage for reliable detection of expressed viruses [Bukur *et al.*, manuscript in preparation].

### HLA types

We used seq2HLA v2.2 [6] to determine the 4-digit HLA type from the RNA-Seq reads. seq2HLA produces accurate 2-digit calls [15] and 4-digit calls [6]. Where available, we also include the HLA typing data determined by Adams *et al.* [16] (Table 1), in which the HLA Class I and Class II genotypes of the NCI-60 cell lines were determined using sequence-based typing (SBT), a standard assay for HLA typing involving a targeted PCR amplification of genomic DNA in the HLA locus.

### Neo-epitope catalog

Using the determined 4-digit HLA Class I alleles and non-synonymous single nucleotide variants (nsSNVs), cell line-specific HLA Class I neo-epitope candidates are determined as described previously [6], with the exception of using NetMHCpan v2.8 [17] as HLA binding prediction tool and using the percentile rank as measure of the best epitope selection instead of IC50. Similarly, we determine the HLA Class II neo-epitopes for these nsSNVs using NetMHCIIpan v3.0 [18] and the cell line HLA-DRB1 type. If a mutation gives rise to multiple equally prioritized neo-antigens (that is, they have the same minimal percentile rank), all results are reported. Only predicted neo-antigens with a percentile rank less than 32 are reported.

### Data storage and web access

To store, integrate, display, and interrogate the data, we developed a platform based on Django, a python web framework (http://www.ncbi.nlm.nih.gov/genome/viruses/). Within Django, data tables are described in Python models that are database agnostic, allowing one to run the system on, for example, SQLite or PostgreSQL. For performance reasons, the TCLP runs on PostgreSQL in conjunction with the webproxy NGINX and memcache for caching web requests.

We designed several models that describe the different data elements, describe their relationships and hold the data. Within Django, apps divide functionally different data models. In our design, the main separation is between the Core, Ontology, and Molecular apps. As the name suggests, the Core app provides the core functionality of the platform and the associated models store the basic sample information, including name and identifier. The Ontology app holds the data for the ontology-based annotation, including disease and tissue. The Molecular app stores the somatic mutations, gene expression values, and the sample HLA types.

## Utility and discussion

### Web portal

**Fig. 1:**
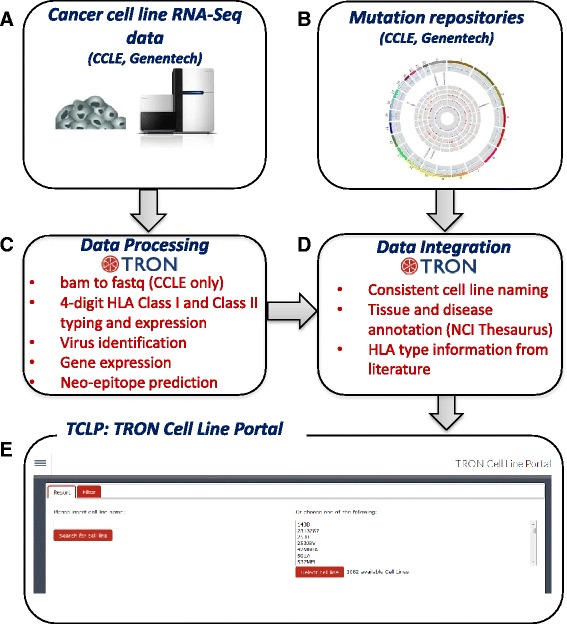
Data integration and computational workflow. RNA-Seq data from 1,083 human cancer cell lines is downloaded from CCLE and Genentech (**a**) and mutation information for the cell lines is retrieved (**b**). The RNA-Seq reads are processed by our in-house pipeline (**c**), consisting of HLA typing and quantification, virus identification, gene expression analysis, and neo-epitope prediction. These data are integrated using consistent cell line names as primary identifier and annotate tissue and disease information using the onotology NCI Thesaurus (**d**). The results are freely accessible in the TRON Cell Line Portal (**e**) at http://celllines.tron-mainz.de

Using our pipeline (Fig. 1), we processed RNA-Seq data from 1,082 human cancer cell lines, generating HLA type and quantification, virus identification and gene expression, and retrieved cell line mutations [4, 5]. The outcome of this pipeline is freely accessible in the TRON Cell Line Portal at http://celllines.tron-mainz.de.

**Fig. 2:**
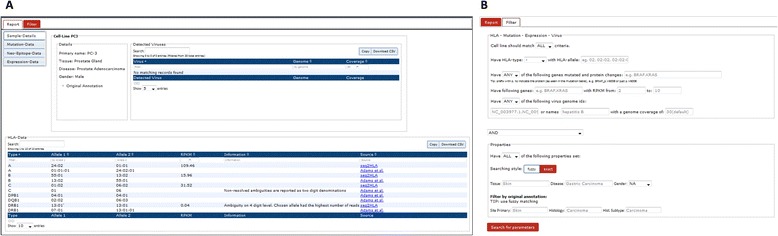
The TRON Cell Line portal (TCLP) offers two main views. **a** The sample information page provides the information of the selected cell line. **b** The advanced search functionality allows the search by a combination and exclusion of criteria

**Fig. 3:**
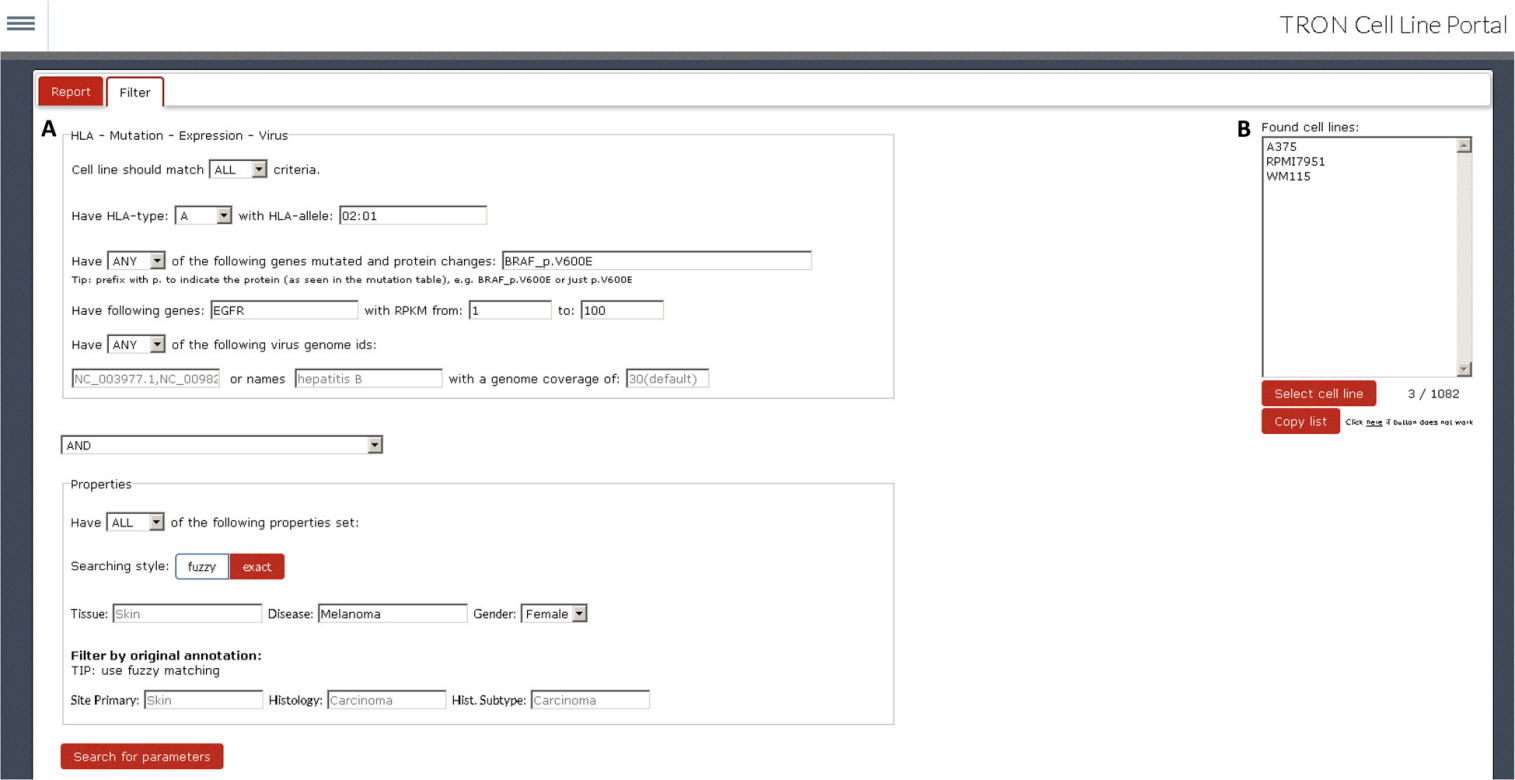
Example search: (**a**) ‘Show me all melanoma cell lines, that (i) are HLA-A*02:01 positive, (ii) express EGFR (between 1 and 1000 RPKM), (iii) have a BRAF p.V600E mutation and (iv) are derived from a female donor. **b** This search reveals three cell lines

The user web interface offers two main views, the sample information page (Fig. 2a) and the advanced search functionality (Fig. 2b). The sample information page provides information about the selected cell line. Through a tab-based interface, tables display tissue and disease type, all linked mutations, gene expression values, detected HLA types, and virus expression. The second view provides advanced search functionality, allowing one to search by a combination and exclusion of criteria. For example, the portal can easily execute the following query: ‘Show me all melanoma cell lines that are (i) HLA-A*02:01 positive, (ii) express EGFR, (iii) have a BRAF p.V600E mutation, and (iv) are annotated as female’. Translating this in the search form, we specify HLA type ‘A’ with allele ‘02:01’, have mutated gene ‘BRAF_p.V600E’, have gene expressed ‘EGFR’ with RPKM from 1 to 100 RPKM, leaving the virus name field empty and do a ‘ALL and fuzzy’ search on the properties to find cell lines annotated as ‘Female’ and have the keyword ‘Melanoma’ in their disease description (Fig. 3a). The cell lines A375, RPMI7951, and WM115 are returned (Fig. 3b). Alternatively, search criteria can also be logically negated, for example, searching for all female melanoma samples that do not have the HLA type A*02:01.

In addition to the user interface, we provide an API based on the Django REST Framework (http://www.django-rest-framework.org/). This provides the user direct access to underlying data models and bulk data retrieval. The user interface relies on and interacts with this API; advanced users can thus discover the available entry points or alternatively browse the API page at http://celllines.tron-mainz.de/api. Additional file 1 shows an example python script to retrieve data using this API.

### HLA type and expression

Knowledge of a cell lines HLA type and HLA expression is critical for immunologic and cancer research and therapeutic development. As an example, in cancer immunotherapy, when developing a vaccine targeting specific mutations presented on a patients HLA allele [19], one might want to use a cancer cell expressing HLA-A*02:01 to identify mutation bearing neo-epitopes presented on HLA [6] and test T-cell activity [20]. In addition, the HLA type of a cell line can be regarded as a molecular identifier [21] and thus HLA typing can be utilized as sample barcode to detect mislabeled or contaminated samples [6].

To our knowledge, this is the largest catalog of HLA type and expression annotated cancer cell lines. Using paired-end RNA-Seq samples from 1,082 cancer cell lines, we determined the 4-digit HLA Class I and Class II type and HLA expression using the tool seq2HLA [6, 15]. When available, HLA typing data from literature are integrated. Figure 2a shows results for the prostate adenocarcinoma cell line PC-3.The HLA Class I type is HLA-A*24:01, HLA-A*01:01, HLA-B*13:02, HLA-B*55:01, HLA-C*01:02, and HLA-C*06:02, consistent with the sequence-based typing (SBT) from Adams *et al.* [16]. In case of HLA-C, the latter only provides 2-digit types, whereas seq2HLA provides the 4-digit HLA type, which is necessary for applications, such as HLA binding predictions [17]. Among HLA Class I allele in PC-3 cells, HLA-A shows the highest (109 RPKM) and HLA-B the lowest expression (16 RPKM). PC-3 expresses HLA Class II alleles at very low levels: HLA-DRB1*13:01 could be correctly identified despite the very small number of mapped reads (0.04 RPKM) while no reads were associated with other HLA Class II alleles.

### Detected viruses

Infections or contaminations of cell lines by viruses can be determined by the presence of viral sequences. As an example, Additional file 2: Figure S1 shows the report for the liver carcinoma cell line PLC/PRF/5 including the determined HLA type and the detected viruses. Here, concordant to the information from the American Type Culture Collection (ATCC), the Hepatitis B virus (HBV) genome is reported. The coverage of above 90 % shows that most of the HBV genome is expressed as mRNA. HBV infection is related to the onset of hepatocellular carcinoma [22] and thus this cell line may act as a model for this cancer entity in terms of HBV infection. Additionally, the Human endogenous retrovirus K113 (HERV-K113) is reported, the only HERV (human endogenous retrovirus) genome present in this database. HERV-K113 is present in many human genomes and is known to express mRNA and even proteins [23, 24].

In addition of identifying new or already known cancer-related virus infections, contaminations can be detected. We find evidence (90 % genome coverage) of murine type c retrovirus in the transcriptome of the bladder urothelial carcinoma cell line 253JBV, which might have confounding effects on experiments [25].

### Mutations

The portal integrates mutation information for the analyzed cell lines from CCLE [4] and Klijn *et al.* [5]. For each mutation, annotations are displayed, such as the affected gene, the position in the genome, the type (for example, substitution), the effect (for example, missense or intron), and the influence on the protein sequence (for example, p.Y58F means, that the Tyrosine residue at position 58 is substituted by a Phenylalanine). In addition, we provide links to the webpage of this entry at the respective source, CCLE or Genentech, and a link to the ‘Drug Gene Interaction Database’, which identifies relationships between mutated genes and drugs [26].

### Neo-epitope catalog

**Fig. 4:**
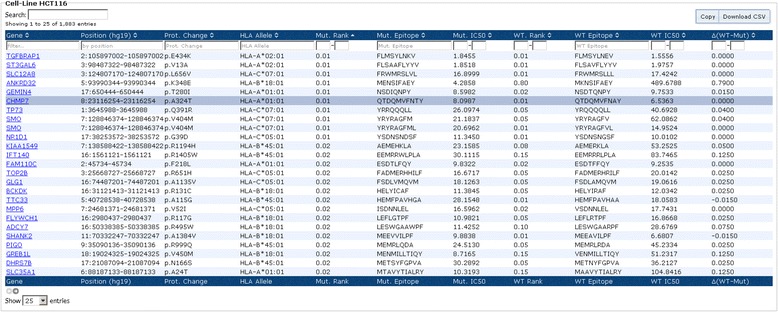
Neo-epitope catalog of HCT116. Columns 1 to 3 describe the mutation, columns 4 to 7 show the HLA allele, the percentile rank, the sequence, and the IC50 of the predicted strongest binding neo-epitope, respectively. Columns 8 to 11 show information for the corresponding wild-type sequence. The marked row is the neo-epitope eluted and identified by mass spectrometry [27]

Using the determined HLA Class I and Class II types in conjunction with the mutations enabled us to define a catalog of HLA Class I and Class II neo-epitope candidates. Figure 4 shows the neo-epitope catalog for colon carcinoma cell line HCT116, sorted from strong to weak binding. The columns 1 to 3 describe the mutation and columns 4 to 7 show the HLA allele, the percentile rank, the sequence, and the IC50 of the predicted strongest binding neo-epitope, respectively. Columns 8 to 11 show information for the corresponding wild-type sequence.

Such a list can be input for experiments searching for tumor HLA-ligands. As an example, Bassani-Sternberg *et al.* [27] recently eluted HLA ligands from HCT116 cells, followed by mass spectrometry profile, and found several mutation-containing ligands, which are listed in the neo-epitope catalog, such as QTDQMVFNTY with a predicted strong binding affinity (rank: 0.01, IC50: 8 nM, marked row in Fig. 4).

### Gene expression

The TCLP allows searching for and listing gene expression values from a selected cell line. The table enables the user to filter via the gene name or to define a RPKM value range. The table dynamically changes its content to display only the data fulfilling the given criteria. The gene name is linked to the NCBI platform for additional gene information. All expression data of the current cell line can be downloaded via a download button at the top of the table or through the corresponding API.

## Conclusion

Cell lines are critical model systems but cell line annotations have been heterogeneous and sparse. Here, we collected and annotated existing public cell line information with ontologies. With internally available computational pipelines, we reprocessed public raw data, including RNA-Seq datasets of 1,082 cancer cell lines, to generate novel annotations including HLA type, HLA expression, HLA Class I and Class II neo-epitope candidates, gene expression, and expressed viruses. Integrating the multiple annotations in one platform with an interactive interface and advanced search capabilities, researchers can effectively identify cell lines for their experiments and targets for therapeutic development.

## Availability and requirements

The TRON Cell Line Portal is freely accessible at http://celllines.tron-mainz.de.

## Additional files

Additional file 1:
**‘fetch_expression_data.py’ is an example python script showing how to use the TCLP API programmatically.** Example calls how to retrieve gene expression and HLA type data. (PY 2 kb) Additional file 2: Figure S1. Virus identification. HBV is expressed in the liver carcinoma cell line PLC/PRF/5. (PPTX 180 kb)

## Abbreviations

API: application programming interface
ATCC: American Type Culture Collection
CCLE: Cancer Cell Line Encyclopedia
HBV: the Hepatitis B virus
HLA: human leukocyte antigen
NCI: National Cancer Institute
NGS: next generation sequencing
RPKM: reads per kilobase of exon per million mapped reads
SBT: sequence-based typings
