# Supplementary figures and images for: TCLP: an online cancer cell line catalogue integrating HLA type, predicted neo-epitopes, virus and gene expression
Source: Genome Med. 2015 Nov 20;7:118. doi: 10.1186/s13073-015-0240-5 (PMC4653878; doi:10.1186/s13073-015-0240-5)

## Slide 1
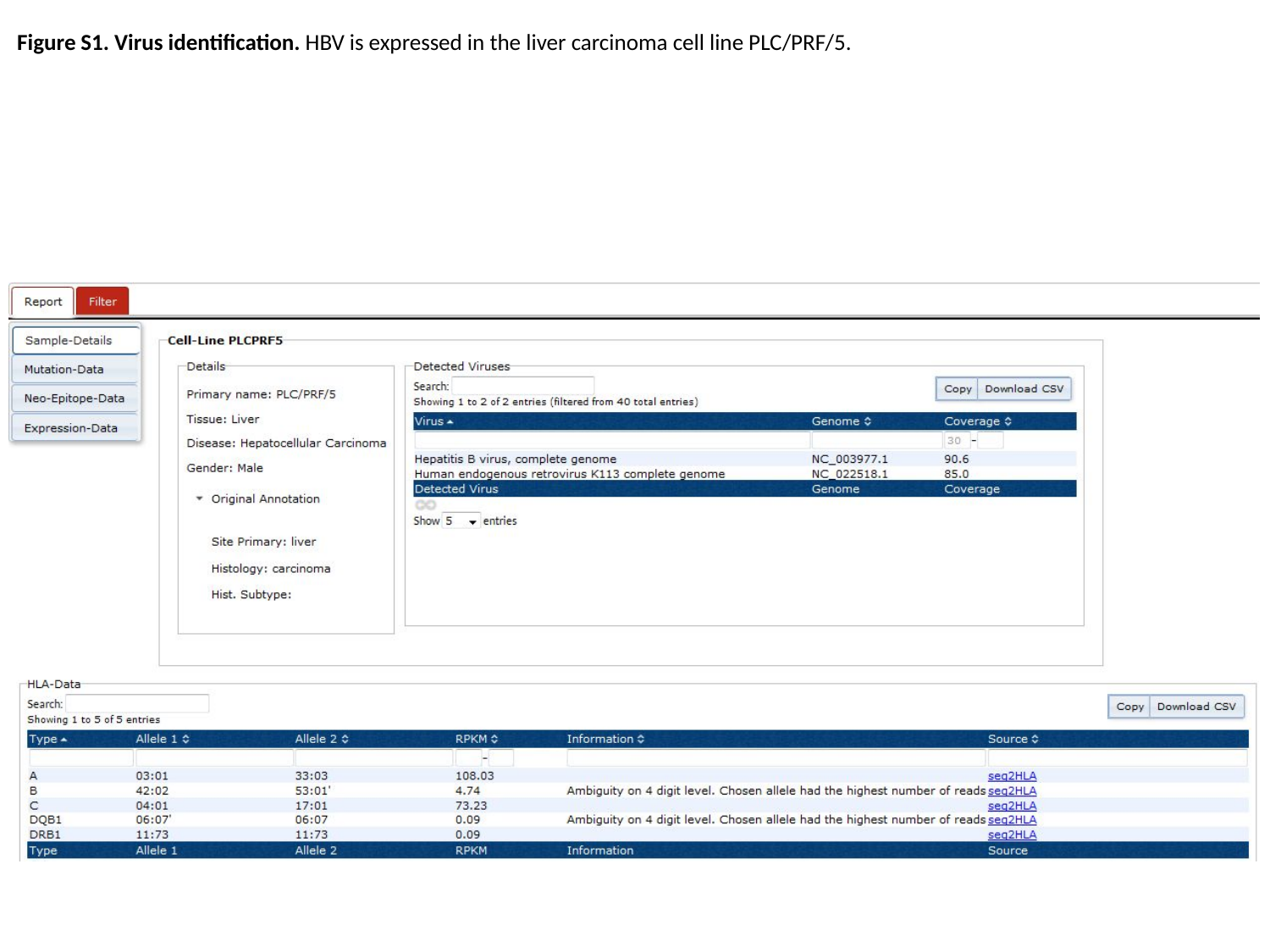

Figure S1. Virus identification. HBV is expressed in the liver carcinoma cell line PLC/PRF/5.

Supplement: Additional file 2: Figure S1. — Virus identification. HBV is expressed in the liver carcinoma cell line PLC/PRF/5. (PPTX 180 kb) [file 13073_2015_240_MOESM2_ESM.pptx]
